# Effects of Baduanjin on glucose and lipid metabolism in diabetic patients: A protocol for systematic review and meta-analysis

**DOI:** 10.1097/MD.0000000000023532

**Published:** 2021-01-29

**Authors:** Qiaojun Ma, Hanzhang Li, Yinping Gao, Yalan Zou

**Affiliations:** a Jiuquan Center for Disease Control and Prevention, Jiuquan; b Second Peoples Hospital of Qingyang City, Gansu Province, Qingyang, Gansu province, China.

**Keywords:** Baduanjin, diabetes mellitus, glucose and lipid metabolism, meta-analysis, protocol, randomized controlled trials

## Abstract

**Background:**

Baduanjin is an ancient technique of physical and breathing exercises (Dao Yin). This technique is divided into eight sections and each section is a motion, so it is called “Baduanjin”. It is practice without equipment, simple and easy to learn, whose effect is significant leading to good fitness effect. Diabetes mellitus is a chronic metabolic disease. Clinical studies have reported that Baduanjin can affect the metabolism of blood glucose and blood lipid in diabetic patients, but the reported efficacy is different among different studies. Therefore, the study is aimed to systematically evaluate the size and differences of the impact of Baduanjin on the metabolism of glucose and lipid in diabetic patients.

**Methods:**

Retrieved randomized controlled trials(RCTs) on effects of Baduanjin on glucose and lipid metabolism in diabetic patients from PubMed, Web of Science, the Cochrane Library, Embase, CNKI with computer while mutually retrieved the same things from Chinese Clinical Trial Registry(ChiCTR), Google Academic and Baidu Academic. The retrieval time was from their establishment to October 2020. Then 2 researchers independently extracted relevant data and evaluated the quality of the included literatures, and meta-analysis was conducted on the included literatures using RevMan5.3.

**Results:**

This research used outcome indicators like fasting blood glucose, postprandial blood glucose, glycosylated hemoglobin, total cholesterol content and triglyceride content to explore the effect of Baduanjin on glucose and lipid metabolism in diabetic patients specifically.

**Conclusion:**

The research will provide reliable evidence-based proof for Baduanjin improving glucose and lipid metabolism of diabetic patients.

**Ethics and dissemination:**

Private information from individuals will not be published. This systematic review also does not involve endangering participant rights. Ethical approval was not required. The results may be published in a peer-reviewed journal or disseminated at relevant conferences.

**OSF Registration number:**

DOI 10.17605/ OSF.IO/AGJHQ.

## 1 Introduction

Diabetes mellitus is a common chronic metabolic disease that seriously endangers human health, mainly manifested by hyperglycemia and often accompanied by disorders of blood-lipid metabolism.^[1]^ Hyperglycemia and long-term metabolic disorders in diabetic patients can cause damage to tissues and organs throughout the body, leading to serious complications.^[2]^ According to the functional status of insulin, diabetes mellitus is divided into type 1 diabetes and type 2 diabetes, of which about 90% is type 2 diabetes. Type 2 diabetes is a metabolic disease caused by multiple causes and characterized by hyperglycemia. It is commonly seen in middle-aged and elderly people, with insidious onset and no obvious symptoms in the early stage.^[3,4]^ With the acceleration of population aging and the change of people's lifestyle in recent years, the incidence of diabetes mellitus is on the rise.^[5]^ The prevalence of diabetes mellitus throughout the world has caused a huge economic burden on the health care system and has a negative impact on the life quality of individuals.^[6]^

Physical exercise is an important strategy for the prevention and treatment of non-communicable diseases. Therefore, as a non-drug, economical and effective means for the prevention and treatment of diabetes mellitus, it has gradually attracted peoples attention. Baduanjin is a kind of guiding art in ancient China, which has the effect of regulating the body, qi movement and functions of zang-fu viscera, dispelling diseases and keeping fit. It is the wealth created by health-keeping experts and martial arts practitioners in past dynasties.^[7]^ Modern clinical practice has proved that Baduanjin plays a therapeutic and rehabilitative role in hypertension, chronic obstructive pulmonary disease (COPD), chronic heart failure, myocardial infarction and etc.^[8–11]^ Baduanjin does not require the use of equipment, and will not be restricted because of the site. It is a long-term, low-intensity aerobic exercise.^[12]^ More and more studies have shown that Baduanjin has certain efficacy in controlling blood glucose and blood lipid levels in diabetic patients improving patients quality of life.^[13–15]^ Studies have shown that Baduanjin prevents and improves hyperlipidemia mainly by promoting glucose decomposition and consumption, improving psychological state, relieving anxiety and depression, spreading knowledge of keeping health and changing patients diet custom.^[16]^ However, as for how Baduanjin affects glucose and lipid metabolism of diabetic patients, as well as the size and difference of its influence, there are differences among different research results. In this study, through a systematic review of published RCTS, the evidence-based basis of Baduanjin's influence on glucose and lipid metabolism of diabetic patients was obtained.

## 2 Methods

### 2.1 Protocol register

This protocol of systematic review and meta-analysis has been drafted under the guidance of the preferred reporting items for systematic reviews and meta-analysis protocols (PRISMA-P). Moreover, it has been registered on the open science framework (OSF) on October 24, 2020. (registration number: DOI 10.17605/OSF.IO / AGJHQ).

### 2.2 Ethics

Since the protocol does not involve the patient's personal information, it is not involved in ethical issues and approval from the ethics committee is not required.

### 2.3 Eligibility criteria

#### 2.3.1 Types of studies

We will collect all available RCTs on the effects of Baduanjin on glucose and lipid metabolism in patients with diabetes mellitus, regardless of blinding, publication status, or region, but the language is limited to Chinese and English.

#### 2.3.2 Research objects

Patients who is definitely diagnosed as diabetes mellitus, regardless of nationality, race, age, sex, region, course of disease, or blood glucose level.

#### 2.3.3 Intervention measures

The treatment group was treated with Baduanjin plus western medicine, including the treatment with Baduanjin alone. There was no restriction on the types and schools of Baduanjin. The control group only used western medicine treatment and the type, dose and the course of treatment were not limited.

#### 2.3.4 Outcome indicators

1. Glucose metabolism level: ①fasting blood glucose (FPG); ②2 hour postprandial blood glucose (P2hPG); ③glycosylated haemoglobin (HbA1c);
2. Lipid metabolism level: ①total cholesterol (TC); ②triglyceride (TG); ③low density lipoprotein (LDL); ④high density lipoprotein (HDL).

### 2.4 Exclusion criteria

1. Studies that can not obtain the full text.
2. Studies published as abstracts, with uncomplete data and unable to obtain data after contacting the author;
3. Studies published repeatedly except the one who has the intact data and best quality.
4. Studies with no relevant outcome indicators.
5. Treatment or control group use other exercises or Chinese medical therapy like Tai Chi, yoga, acupuncture, etc.

### 2.5 Retrieval strategy

“Ba Duan Jin (Baduanjin)”, “Tang Niao Bing (Diabetes mellitus)”, “Tang Zhi Dai Xie (Glucose and lipid metabolism)”, and other Chinese terms were searched on Chinese database like CNKI, VIP, Wanfang Data Knowledge service platform, and China Biomedical Database. “Eight trigrams boxing”, “Baduanjin” and “Diabetes Mellitus” were searched as English searching terms in PubMed, Web of Science, the Cochrane Library and Embase, at the same time, we searched mutually in Google Academic, Baidu Academic and Chinese Clinical Trial Registry (ChiCTR). The searching time was from their establishment to October 2020. All above is for collecting domestic and foreign literatures on the effect of Baduanjin on glucose and lipid metabolism in diabetic patients. Taking PubMed as an example, its retrieval strategy was shown in the Table 1.

**Table 1:** Retrieval strategy in PubMed.

Number	Search terms
#1	eight trigrams boxing [Title/Abstract]
#2	Baduanjin [Title/Abstract]
#3	eight-sectioned exercise [Title/Abstract]
#4	eight sections brocade [Title/Abstract]
#5	Eight duan Jingong [Title/Abstract]
#6	#1 OR #2 OR #3 OR #4 OR #5
#7	Diabetes Mellitus [MeSH]
#8	Diabetes Mellitus [Title/Abstract]
#9	Glucose Metabolism Disorders [Title/Abstract]
#10	Diabetics [Title/Abstract]
#11	glucolipid metabolism [Title/Abstract]
#12	#7OR #8 OR #9 OR #10 OR #11
#13	#6AND #12

### 2.6 Data screening and extraction

**Figure 1:**
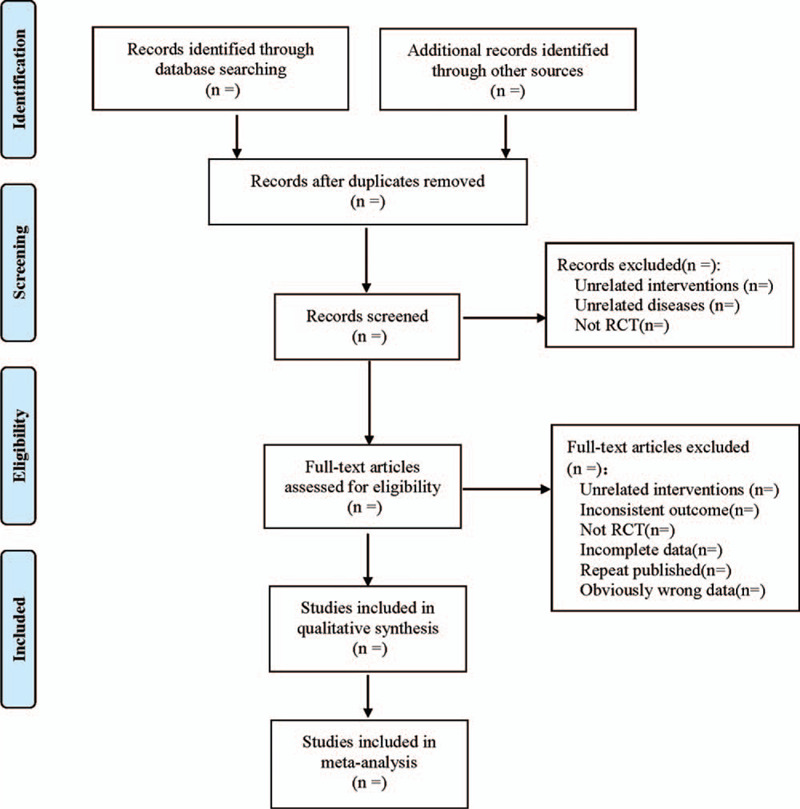
Flow diagram.

Referring to Cochrane Handbook for Systematic Reviews of Interventions, 2 researchers used EndNote X7 to independently screen according to inclusion and exclusion criteria. Firstly, the literature of each data was imported into EndNote X7 so, and then repeated literatures were removed. Then, the title and abstract were read for preliminary screening. After the preliminary screening, the full text was further read for screening, and finally qualified studies were included for systematic evaluation and meta-analysis. Marked down reasons for exclusion for each article during the filtering process. The 2 researchers checked the screening results and discussed the results with the third researcher for those studies that were difficult to be included due to differences. At the same time, Excel 2013 was used to extract relevant information, including: ①First author of the literature, year of publication; ②Basic information of the subjects: age, sex, height, weight, course of disease and complications; ③Intervention methods of the treatment group and the control group: The treatment group was treated with Baduanjin exercise or Baduanjin combined with western medicine, while the control group was treated with western medicine only. Their frequency, dosages and courses; ④ Outcome indicators: FPG, P2hPG, HbA1c, TC, TG, HDL, etc. ⑤The quality evaluation factors of the literatures; ⑥Adverse events and follow-up. The process of literature filtering was shown in Figure 1.

### 2.7 Literature quality evaluation

Use the Cochrane collaborations tool for assessing risk of bias to performed risk bias assessment of the included studies. According to the performance of the included literatures in the evaluation items below which are random sequence generation, allocation concealment, blinding of participants and personnel, blinding of outcome assessment, incomplete outcome data, selective reporting and other bias, the 2 researchers will give low risk, unclear and high risk judgments one by one and cross-check after completion respectively. In case of any disagreement, discussion will be carried out. If no agreement can be reached, discussion will be made with the researchers of the third party.

### 2.8 Statistical analysis

The RevMan 5.3 software was used for meta-analysis. Relative risk (RR) was selected as the statistic for the dichotomous variable. For continuous variables, weighted mean difference (WMD) was selected when the tools and units of measurement indicators were the same, standardized mean difference (SMD) was selected as effect value if tools or units of measurement were different. And all above were represented by 95% confidence interval (CI). Heterogeneity was determined by χ^2^ and *I*^*2*^.If *P* ≥ .1and *I*^*2*^ ≤ 50%, which meant low heterogeneity, we used fixed-effect model to do meta-analysis; If *P* < .1 and *I*^*2*^ > 50%, which indicated inter-study heterogeneity, the source of heterogeneity would be explored, and then the clinical heterogeneity was dealt with through subgroup analysis. If no significant clinical or methodological heterogeneity were found, statistical heterogeneity was considered and the random-effect model was used for analysis. If the clinical heterogeneity was too obvious and the subgroup analysis could not be performed, descriptive analysis was performed instead of making meta-analysis.

#### 2.8.1 Dealing with missing data

If there is missing data in the article, contact the first author or corresponding author via email or phone to obtain accurate data. If the author cannot be contacted, or the author has lost relevant data, descriptive analysis will be conducted instead of meta-analysis.

#### 2.8.2 Subgroup analysis

Subgroup analysis was performed according to that if use Baduanjin alone or Baduanjin combined with western medicine in the treatment group. Subgroup analysis was performed according to the age of the patients. Subgroup analysis was performed according to the type of diabetes mellitus. Subgroup analysis was performed according to the courses of treatment.

#### 2.8.3 Sensitivity analysis

In order to determine the stability of outcome indicators, sensitivity analysis was used to analyze each outcome indicator.

#### 2.8.4 Assessment of reporting biases

Funnel plots were used to assess publication bias if no fewer than 10 studies were included in an outcome measure. Otherwise, Egger and Begg test were used for the evaluation of potential publication bias.

## 3 Discussion

Diabetes mellitus is one of the chronic diseases in developed and developing countries around the world. According to the International Diabetes Federation, the number of diabetics worldwide will reach 693 million by 2045.^[17]^ Diabetes mellitus has a long course of disease and many complications, which cause a great burden on families. In developed countries, two-thirds of patients has poor disease management.^[18]^ Common complications of diabetes mellitus include cardiovascular and cerebrovascular diseases, diabetic foot, diabetic gastroparesis, etc., all of which are common causes of death and disability in diabetic patients.^[19]^ Diabetes mellitus is divided into type 1 and type 2. Insulin resistance is considered to be the main cause of type 2 diabetes, and abnormal glucose and lipid metabolism is a symptom of insulin resistance.^[20]^ The symptoms of diabetic patients are mainly polydipsia, polyphagia, and polyuria, etc. With the aggravation of the disease, the weight of the patients will gradually decrease and people will become relatively thin, which is more obvious in type 1 diabetes. In type 2 diabetes, the symptoms are mostly systemic fatigue and weight gain before onset. If not treated promptly, weight loss will gradually occur. At present, most of the drugs in western medicine for the treatment of diabetes mellitus are symptomatic treatment with large adverse reactions.^[21]^

In traditional Chinese medicine, diabetes mellitus belongs to the categories of “consumptive thirst (Xiao Ke)”, “consumptive disease (Xu Lao)” and etc. In general treatise on causes and manifestations of all diseases (Zhu Bing Yuan Hou Lun), it is indicated that if consumptive thirst (Xiao Ke) is improperly treated, or if the course of the disease persists indefinitely, it may develop into carbuncle and abscess (Yong Ju) or edema, which are diabetic foot and diabetic nephropathy in modern medicine. Traditional Chinese medicine advocates prevention before disease, which is of great significance for the prevention and treatment of diabetes. The main treatment measures for diabetes mellitus are “5 carriages”, namely, diabetic education, diet control, exercise, drugs and blood glucose monitoring, among which exercise is a necessary means for diabetes mellitus treatment.^[22]^ Studies have shown that exercise can improve the condition of blood lipid and blood pressure of diabetic patients and improve their quality of life.^[23]^ As a non-drug and economical means, Baduanjin has a long history and culture as well as health value. It is simple and easy to learn and suitable for people of all ages. While improving the health of the masses, it also reduces the burden of medical care for the country and the people. Baduanjin exercise can significantly improve endothelium-dependent vasodilation function in type 2 diabetes patients, which is very beneficial to restore the health of patients with diabetes mellitus.^[24]^ The specific reasons are related to the fact that Baduanjin can improve the skin temperature of the Governor Vessel points, increase the Yang Qi of human body, excite the vital gate fire of human body and regulate the running of qi and blood in meridians.^[25]^Studies have shown that Baduanjin can play an anti-diabetes role by regulating the expression of mRNA, IncRNA, and circRNA.^[26]^ Non-medical intervention of physical rehabilitation has a great positive effect on the rehabilitation of diabetic patients, mainly treating the interior to cure the root of diabetes. And where traditional Chinese medical physiotherapy is superior to western medicine treatment is that it has no toxic and side effects at all.^[27]^ However, the clinical efficacy of Baduanjin in the treatment of diabetes mellitus has not been recognized by international authoritative medical organizations. Therefore, we analyzed the RCTs of the specific impact of Baduanjin on glucose and lipid metabolism of diabetic patients, obtained the theoretical basis of improving glucose and lipid metabolism of diabetic patients with Baduanjin and objectively evaluated the impact of Baduanjin on diabetic patients. However, this study also has some limitations. Due to the special operation of Baduanjin, it is difficult for us to use blind method. Moreover, due to language restrictions, we only searched Chinese and English literatures, which may have some publication bias.

## Author contributions

**Conceptualization:** Hanzhang Li.

**Data collection:** Qiaojun Ma and Hanzhang Li.

**Data curation:** Qiaojun Ma, Hanzhang Li.

**Funding acquisition:** Hanzhang Li.

**Funding support**: Hanzhang Li.

**Investigation:** Qiaojun Ma, Hanzhang Li.

**Literature retrieval:** Qiaojun Ma and Hanzhang Li.

**Software:** Yinping Gao.

**Software operating**: Yinping Gao.

**Supervision:** Yalan Zou.

**Writing – original draft:** Qiaojun Ma, Hanzhang Li.

**Writing – review & editing:** Yinping Gao.

## Abbreviations

Abbreviations: ChiCTR = Chinese Clinical Trial Registry, CI = confidence interval, COPD = chronic obstructive pulmonary disease, FPG = fasting blood glucose, HbA1c = glycosylated hemoglobin, HDL = high density lipoprotein, LDL = low density lipoprotein, P2hPG = 2 hour postprandial blood glucose, PRISMA = the preferred reporting items for systematic reviews and meta-analyses, RCTs = randomized controlled trials, RR = relative risk, SMD = standardized mean difference, TC = total cholesterol, TG = triglyceride, WMD = weighted mean difference.
